# Applying a neoscore in locally advanced rectal cancer is beneficial in predicting local recurrences after surgery

**DOI:** 10.1371/journal.pone.0285709

**Published:** 2023-05-12

**Authors:** Amal Rayan, Ahmed Soliman

**Affiliations:** 1 Clinical Oncology Department, Faculty of Medicine, Assiut University, Assiut, Egypt; 2 General Surgery Department, Faculty of Medicine, Assiut University, Assiut, Egypt; Osaka Medical Center for Cancer and Cardiovascular Diseases, JAPAN

## Abstract

**Background and aim:**

The current study was undertaken to provide more detailed prognostic models for early prediction of local recurrences and local recurrence free survival (RFS) using different radiologic and pathologic features of locally advanced rectal carcinomas treated with neoadjuvant chemoradiation (CRT).

**Methods:**

One hundred patients with locally advanced rectal carcinomas decided to receive neoadjuvant CRT were retrospectively recruited, Hazard ratios (HR) were determined in the two cox regression models and only significant ratios were considered for pointing, Models were built to determine their important effects of different predictors including: pathologic T (T), pathologic N (N), grade (G), clinical stage (cTNM), site (S), perineural invasion (PNI), and response to CRT (R) on 3-year RFS, goodness of performance of each model was measured by Harrell’s C index.

**Results:**

HR of 1^st^ group of models: T+N, T+N+G, T+N+G+S, T+N+G+S+PNI, and T+N+G+S+PNI+R were summated and categorized into scores, these scores were significantly correlated with the risk of recurrence (Somer’s D = 0.5, *p*<0.0001) & Harrell’s C index = 0.751, (Somer’s D = 0.6, *p*<0.0001) & its Harrell’s C index = 0.794, (Somer’s D = 0.7, *p*<0.0001) & C index = 0.826, Somer’s D = 0.7, *p*<0.0001) & C index = 0.827, and (Somer’s D = 0.7, *p*<0.0001) & C index = 0.843 respectively. The 2^nd^ group of models including: cTNM stage, cTNM+G, cTNM+G+S, cTNM+G+S+PNI, cTNM+G+S+PNI+R scores which were significantly correlated with the HR of LRR (Somer’s D = 0.2, 0.5, 0.6, 0.6, & 0.6 respectively), (*p* = 0.006, <0.0001, <0.0001, <0.0001, <0.0001 respectively), the corresponding Harrell’s C indices were 0.595, 0.743, 0.782, 0.795, & 0.813 respectively.

**Conclusion:**

We propose that the addition of biologic factors to staging of rectal cancer provide precise stratification and association with local recurrences in patients received preoperative CRT.

## Introduction

Colorectal cancers are the third most commonly diagnosed cancers among men and women in USA, one third of these cancers are rectal cancers [1]. According to GLOBOCAN 2018 data, colorectal cancer (CRC) is the third most deadly and fourth most commonly diagnosed cancer in the world [2].

The close proximity of the rectum to pelvic organs with absence of serosal covering surrounding the rectum, and restricted surgical access by the pelvic cavity, so the loco-regional recurrence (LRR) rate in rectal cancer is relatively high after surgery alone; as previously reported the risk of LRR of T3 or T4 ±N+ rectal cancer without adjuvant therapy, ranged from 15 to 65% [3, 4].

To improve the local control after conventional surgery, radiotherapy (RT) has been utilized. Several randomized controlled trials (RCTs) showed that peri-operative RT could reduce the LRR rate and improve the overall survival (OS) rate, establishing the standard multimodality treatment of rectal cancer utilizing preoperative concurrent chemoradiation (CRT) followed by surgery and postoperative chemotherapy in stages II and III as a standard of care [5–9].

However, it is unclear whether preoperative CRT is superior to postoperative CRT for OS. Meanwhile, several pilot studies suggested that preoperative chemotherapy combined with total mesorectal excision (TME) instead of preoperative CRT plus TME might be more beneficial in terms of OS and saved patients from adverse side effects related to RT [10].

The goal of staging is to inform the prognosis and guide for the proper treatment plan of patients, preoperative staging is based mainly on imaging and endorectal ultrasound and the choice for CRT depends on this staging, however following surgery and determination of pathologic staging, rectal cancers may be upstaged. Whether clinical stage, pathologic stage, and grades of tumor necrosis greatly influence local recurrence are a matter of debate.

Clinical and radiological features of locally advanced rectal carcinoma have limited ability to predict local recurrences following neoadjuvant CRT, and the pathologic response (pCR) to neoadjuvant treatment may predict a survival advantage: both disease free survival and overall survival [11–13]. Molecular profiling holds the greatest potential to predict pCR but its adoption will require greater concordance between cohorts for the biomarkers currently under investigation [14].

Currently, there are 5 different classifications for evaluating the pathologic response to neoadjuvant CRT, and the American Joint Committee on Cancer classification is considered the standard one [15, 16]. It involves the following categories: (R0) complete response, when no tumor cells are observed, (R1) moderate response, when only small tumor nests or single cells are observed, (R2) minimal response, when there is residual cancer but predominant fibrosis, and (R3) poor response when tumor regression is minimal or null.

The 12-study meta-analysis published in 2012 [17], reported a 5-year local recurrence rate of 0% in patients achieved pCR to neoadjuvant CRT. The overall weighted average of the LRR in these studies was 7%. Furthermore, patients with pCR were 4 times less probable to develop local recurrence compared with those having a partial response or no response.

Bioscore incorporating grade, HER2neu status and estrogen receptor (ER) status in addition to either pathologic stage or T+N stages provided more refined stratification of breast cancer patients who underwent radical surgery [18]. Moreover, neo-bioscore utilizing clinical pathologic stage, ER status and grading facilitated more refined categorization into prognostic subgroups than clinical or final pathologic stage alone and the addition of HER2neu status provided improved stratification of patients with respect to prognosis [19].

The current study was undertaken to provide more detailed prognostic models for early prediction of local recurrences and local recurrence free survival using different radiologic and pathologic features of locally advanced rectal carcinomas treated with neoadjuvant CRT.

## Methods

One hundred patients with locally advanced rectal carcinomas treated by neoadjuvant chemoradiation (capecitabine 850 mg/m^2^/dose twice daily given concurrently with 3D-conformal radiotherapy 45–50.4 Gy/25-28 fractions), were retrospectively collected from data registry of clinical oncology department, Assiut University. Clinicopathologic data were recorded including: age, sex, performance status according to (Eastern Cooperative Oncology Group-Performance Status) ECOG scale, pathologic subtypes, pathologic grades, staging, response to neoadjuvant CRT, and time to LRR following surgery, types of surgery (conservative surgery or abdominoperineal resection (APR), all surgeries were done with total mesorectal excision (TME). patients with incomplete data or without TME were excluded. Patients treated during the period from January 2015 to December 2017 were enrolled.

The clinical endpoint in this study was 3-year recurrence free survival (RFS), which was calculated from time of surgery to time of LRR, patients continued for more than 3 years without recurrence and those missed without completing 3 years were considered censored.

Magnetic resonance imaging (MRI), endorectal ultrasound, multisclice CT chest and abdomino-pelvis with contrast were done to determine T and N stages before chemoradiation

Response to CRT was determined through preoperative proctoscopy and biopsy then categorized according to AJCC classification, in addition to pelvic MRI, and endorectal U/S, however, full evaluation of different pathologic responses was mainly determined after surgery.

Univariate analysis and cox regression hazard proportional analysis of different prognostic factors for local relapse free survival including T, N, pathologic stage, pathologic type, grade, (carcinoembryonic antigen) CEA, distance of the tumor from anal verge (≥6 cm or <6 cm), type of surgery (conservative surgery vs. abdominoperineal resection), perineural invasion (PNI), lymphovascular invasion (LVI), and response to neoadjuvant CRT.

Two cox regression analyses were constructed for ordinal and dichotomous variables only, the first one involved pathologic T stages and pathologic N stages and the second one involved cTNM stages, response to neoadjuvant CRT, grade, pathologic type, CEA, site, type of surgery, PNI. These predictors were the significant ones in univariate analysis.

### Modeling and statistics

Hazard ratios (HR) were determined in the two cox regression analyses and only significant ratios were considered for pointing, and the following points were given for ordinal variables: HR; 1.1–3 was given 1 point, HR; 3.1–6 was given 2 points, HR; 6.1–10 was given 3 points, HR> 10 was given 4 points.

While for binary variables 1 point was given for the significant variable, in addition, the reference category in each variable was the first category in all ordinal variables, and the negative category in all binary variables. Non-significant HR in any category of each variable in the proportional HR analyses was pointed 0.

In each model, the range of points and its frequency were determined and scored, provided that no score had a frequency less than 10, then the score of each model was further analyzed using cox regression to determine its significance and x^beta value to run concordance test to detect Harrell’s C index [20] to insure goodness of the model and Somer’s D value of correlation with 3-year RFS; the model to be considered good should be of Harrell’s C value >0.5 and the index was used for comparison between different model performances which ranged from perfect discordance (0,0) to perfect concordance (1,0). Akaike information criterion (AIC) is an estimator of prediction error and relative quality of statistical models where an amount of information is lost with each statistical model, AIC gives us an estimation of the amount of information lost and deals with both the risk of overfitting and underfitting, The 3-year DFS curves of different model scores were drawn by cox regression analysis.

AIC = *2k-2Ln* (*L*) where *k* is the number of estimated parameters in each model; *L* is the maximum value of likelihood function of the model.

Models were built to determine their important effects of different predictors including: pathologic T (T), pathologic N (N), grade (G), clinical stage (cTNM), site (S), PNI, and response to CRT (R) on 3-year RFS.

The first group of models included: T+N, T+N+G, T+N+G+S, T+N+G+S+PNI, and T+N+G+S+PNI+R.

The second group included cTNM, TNM+G, TNM+G+S, TNM+G+S+PNI, and TNM+G+S+PNI+R.

The scores of each model were plotted against 3-year RFS and p-value≤5% was considered significant. Univariate analysis was done through performing the appropriate test according to number of categories in the independent variables; Mann Whitney U-test and Kruskal-Wallis test, all available data were nonparametric based on Shapiro-Wilk (p<0.05), and all data were analyzed using IBM SPSS version 26. The study was approved by ethics committee of faculty of medicine, Assiut university (IRB no; 17100623l), in addition, our ethics committee waived the requirement for informed consent.

## Results

One hundred patients with locally advanced rectal carcinomas received neoadjuvant CRT were followed up for evidence of local recurrence after surgery for a median duration of 24 months (7–36 months), the estimated 3-year local RFS was 29%. Patients continued their follow up without LRR for more than 36 months and those missed during the follow up period were considered censored (24/100), local recurrences and death were reported in 74/100 patients, the Clinicopathologic characteristics and their significant effects on local RFS were shown in Table 1.

**Table 1:** Univariate analysis of Clinicopathologic features of locally advanced cancer rectum.

Characteristics	Descriptive (n = 100)	Univariate analysis *p*-value
Age, mean ±SE range	43.1±1.5 years 17–70 years	0.1
Sex: female/male	45/55	0.04
ECOG-PS		
0	6 (6%)	
1	39 (39%)	0.4
2	52 (52%)	
3	3 (3%)	
Distance from anal verge		
<6 cm	49 (49%)	0.001
≥6 cm	51 (51%)	
Pathologic type		
Adenocarcinoma	70 (70%)	<0.0001
Mucinous carcinoma	13 (13%)	
Signet ring carcinoma	17 (17%)	
Grade		
1	15 (15%)	
2	43 (43%)	<0.0001
3	18 (18%)	
4	24 (24%)	
TNM stage		
1	6(6%)	
2a	10 (10%)	
3a	16(16%)	<0.0001
3b	45 (45%)	
3c	23 (23%)	
LVI		
Positive	39 (39%)	0.3
negative	61 (61%)	
PNI		
Negative	82 (82%)	<0.0001
positive	18 (18%)	
CEA		
Normal	53 (53%)	0.009
High	47 (47%)	
Pathologic response		
R0	22 (22%)	
R1	24 (24%)	<0.0001
R2	31 (31%)	
R3	23 (23%)	
Surgery		
Conservative	60 (60%)	0.001
APR	40 (40%)	

Data were analyzed using Mann Whitney U-test and Kruskal-Wallis test, APR; abdominoperineal resection, CEA; carcinoembryonic antigen, ECOG-PS; Eastern Cooperative Oncology Group-Performance Status.

The results of first proportional hazard analysis using T and N categories with the reference categories used (this table included multivariate analysis of T and N stages to determine their hazard ratios and significant effects on survival): T1 and N0 respectively with their corresponding points were shown in the subsequent table, Table 2.

**Table 2:** 1^st^ proportional hazard analysis of T and N categories on 3-year local RFS and their corresponding points.

	B	SE	Wald	df	*P-value*.	HR	
							points
T1	reference					0	0
T 2	1.061	1.1	0.97	1	0.3	2.889	0
T 3	2.038	1.02	3.97	1	0.046	7.679	3
T 4	2.460	1.04	5.6	1	0.018	11.700	4
N0	reference					0	0
N1a	0.568	0.43	1.8	1	0.2	1.765	0
N1b	0.805	0.4	4.2	1	0.04	2.237	1
N2a	1.253	0.5	7.95	1	0.005	3.502	2
N2b	2.477	0.5	24.3	1	<0.0001	11.908	4

Data analyzed using cox regression test, *F* = 69.95, *p*<0.0001, Wald test ≈ t-test.

The 2^nd^ proportional hazard analysis of the following predictors included: pathologic stage, pathologic type, grade, PNI, CEA, pathologic response to CRT on 3-year RFS and their referent categories with the corresponding points (this test was a multivariate analysis of all significant variables on survival) was illustrated in Table 3.

**Table 3:** 2^nd^ proportional hazard analysis of different predictors on 3-year RFS and their corresponding models.

	B	SE	Wald	df	*p*-value	HR	points
Adenocarcinoma	reference					0	0
Mucinous carcinoma	.083	.415	.122	1	.842	1.086	0
Signet ring carcinoma	.161	.459	10.1	1	.727	1.174	0
Grade 1	reference					0	0
Grade 2	.168	.394	3.79	1	.670	1.182	0
Grade 3	.881	.452	6.61	1	.050	2.413	1
Grade 4	1.491	.580	27.47	1	.010	4.440	2
TNM stage 1	reference					0	0
TNM stage 2	0.333	0.69	0.232	1	0.6	1.395	0
TNM stage 3	1.156	0.593	3.8	1	0.050	3.177	2
Conservative surgery	reference					0	0
APR	0.240	.294	3.85	1	0.414	1.271	0
Site ≥6 cm	reference					0	0
Site <6 cm	0.86	0.31	7.82	1	0.005	2.351	1
PNI negative	reference					0	0
PNI positive	.650	.331	2.25	1	.050	1.915	1
CEA normal	reference					0	0
CEA high	.371	.247	11.69	1	.134	1.449	0
R0 response	reference					0	0
R1 response	-.092-	.438	.838	1	.833	.912	0
R2 response	.382	.418	6.87	1	.360	1.465	0
R3 response	1.198	.457	6.89	1	0.009	3.313	2

Data analyzed using cox regression hazard model, *F* = 113.04, *p*<0.0001, Wald test ≈ t-test.

**Fig 1:**
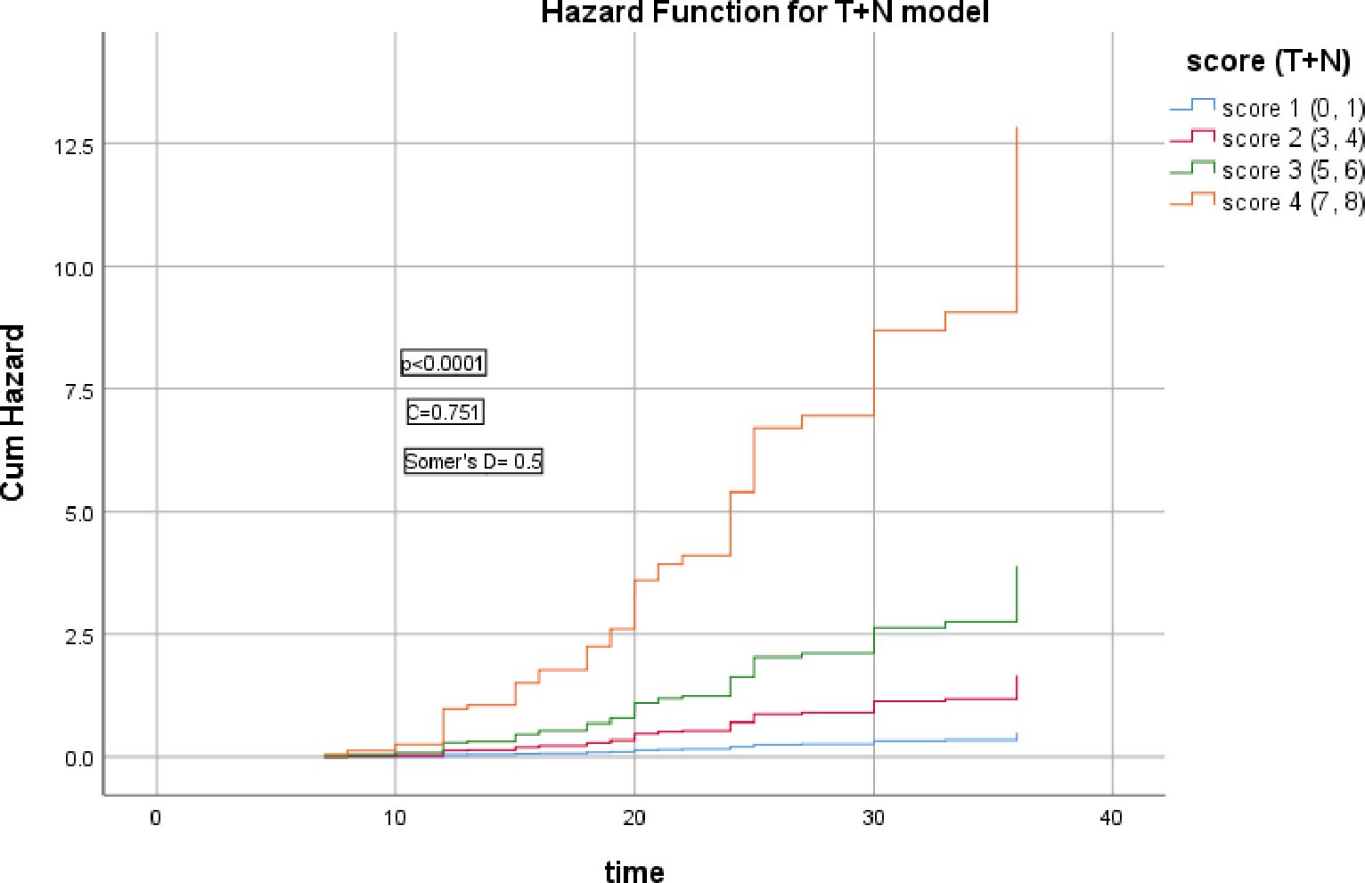
Hazard functions for T+N model and their scoring showed the highest risk of LRR was for score 4.

The points given for the hazard ratios of T+N were summated and categorized into 4 scores, these scores were significantly correlated with the risk of recurrence (Somer’s D = 0.5, *p*<0.0001), Harrell’s C index = 0.751, AIC = 394.49, (Fig 1).

**Fig 2:**
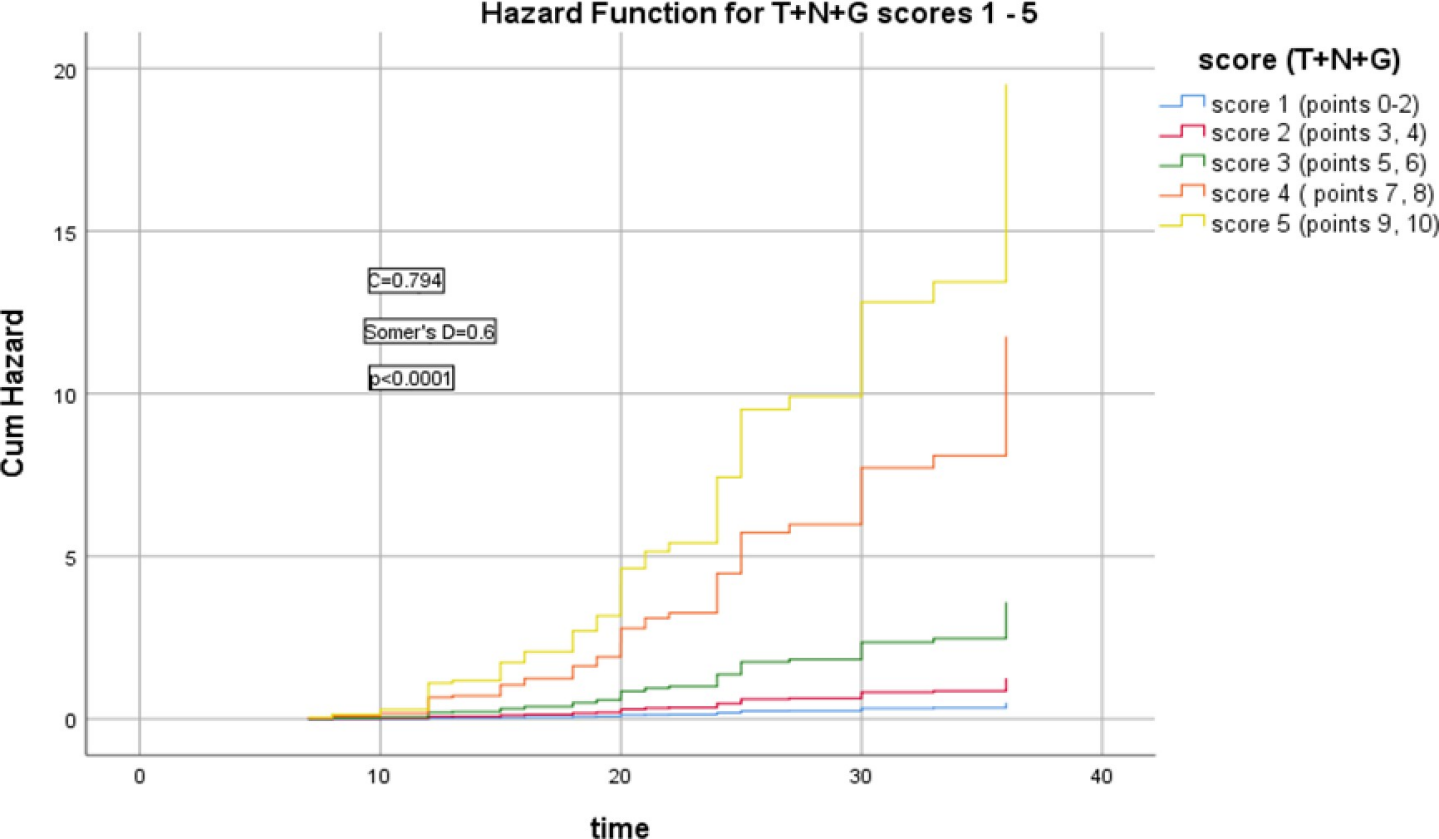
Hazard functions for T+N+G model and their scoring showed the highest risk of LRR was for score 5.

Fig 2 showed the relation between T+N+G model and the HR of LRR (Somer’s D = 0.6, *p*<0.0001), and its Harrell’s C index = 0.794, AIC = 370.828.

**Fig 3:**
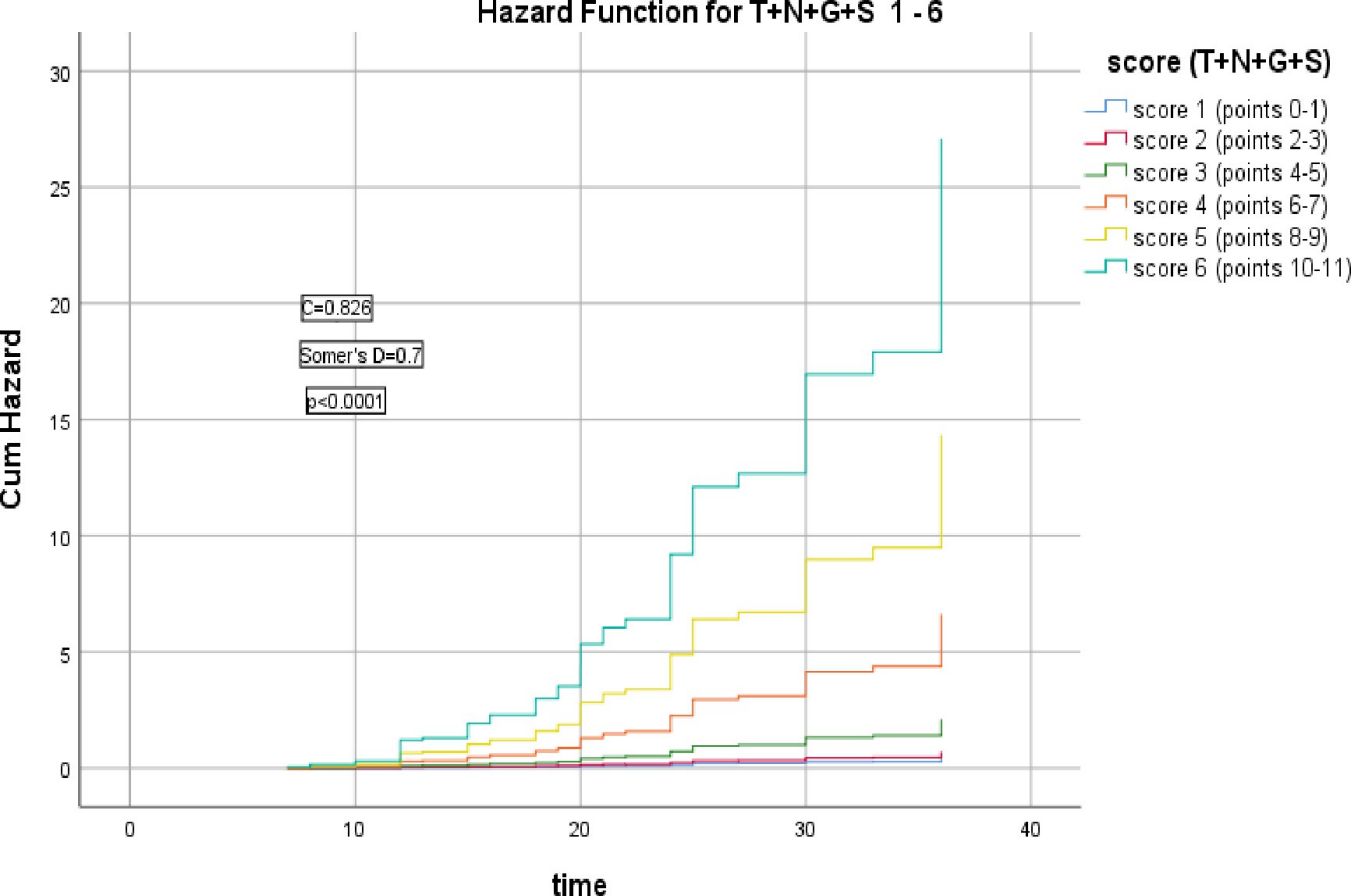
Hazard functions for T+N+G+S model and their scoring showed the highest risk of LRR was for score 6.

There was a significant correlation between T+N+G+S model scores and the HR of LRR and 3-year RFS (Somer’s D = 0.7, *p*<0.0001), C index = 0.826, AIC = 349.244, (Fig 3).

**Fig 4:**
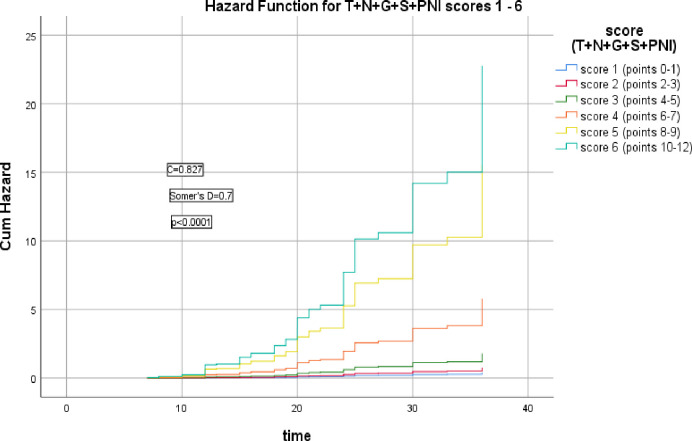
Hazard functions for T+N+G+S+PNI model and their scoring showed the highest risk of LRR was for score 6.

The subsequent figure elicited significant correlation between T+N+G+S+PNI model scores and the HR of LRR and 3-year RFS (Somer’s D = 0.7, p<0.0001), C index = 0.827, AIC = 349.911, (Fig 4).

**Fig 5:**
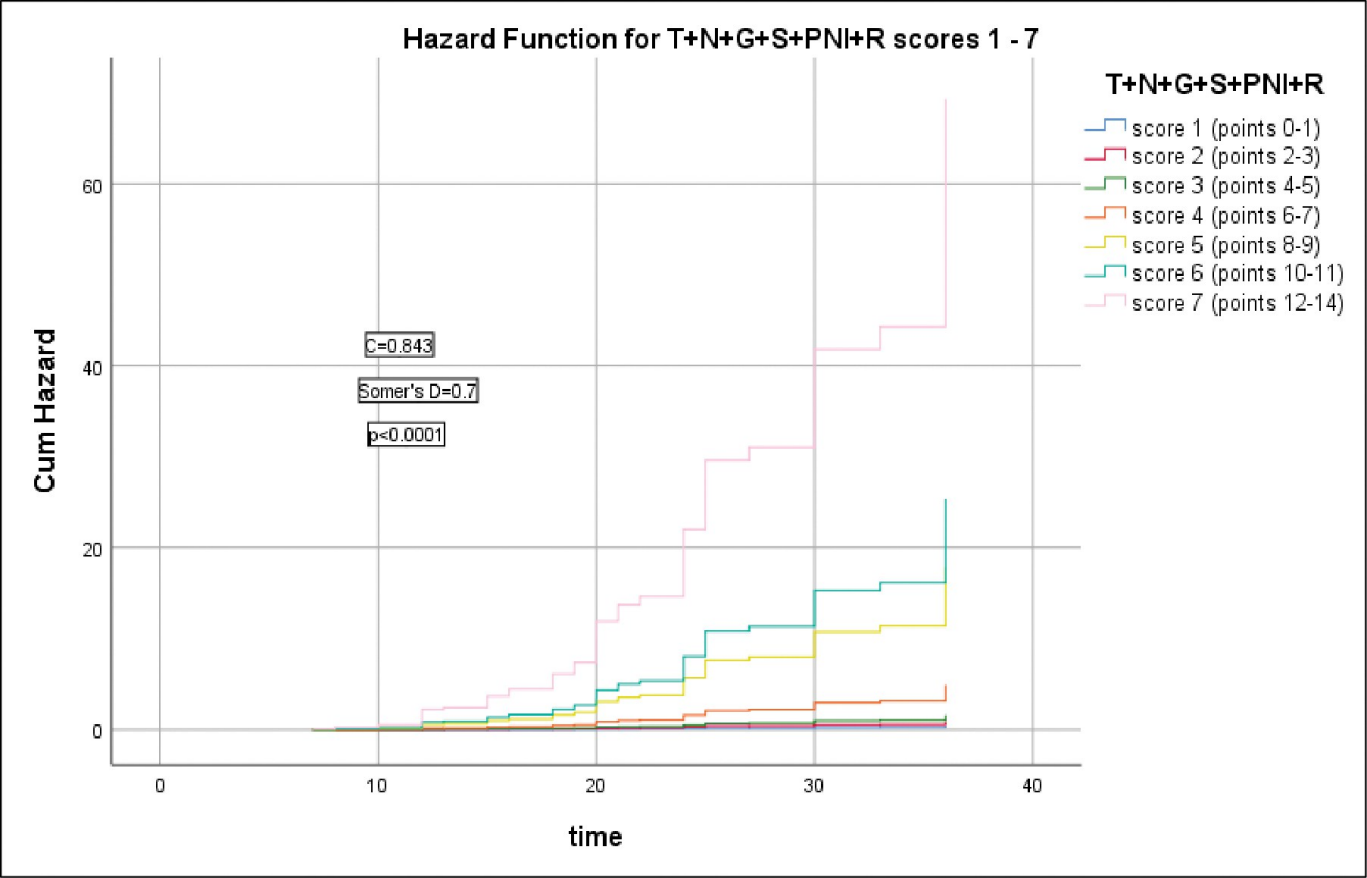
Hazard functions for T+N+G+S+PNI+R model and their scoring showed the highest risk of LRR was for score 7.

T+N+G+S+PNI+R model scores were significantly correlated with the HR of LRR and 3-year RFS (Somer’s D = 0.7, p<0.0001), C index = 0.843, 339.396 and this model had the highest C index and lowest AIC, (Fig 5).

**Fig 6:**
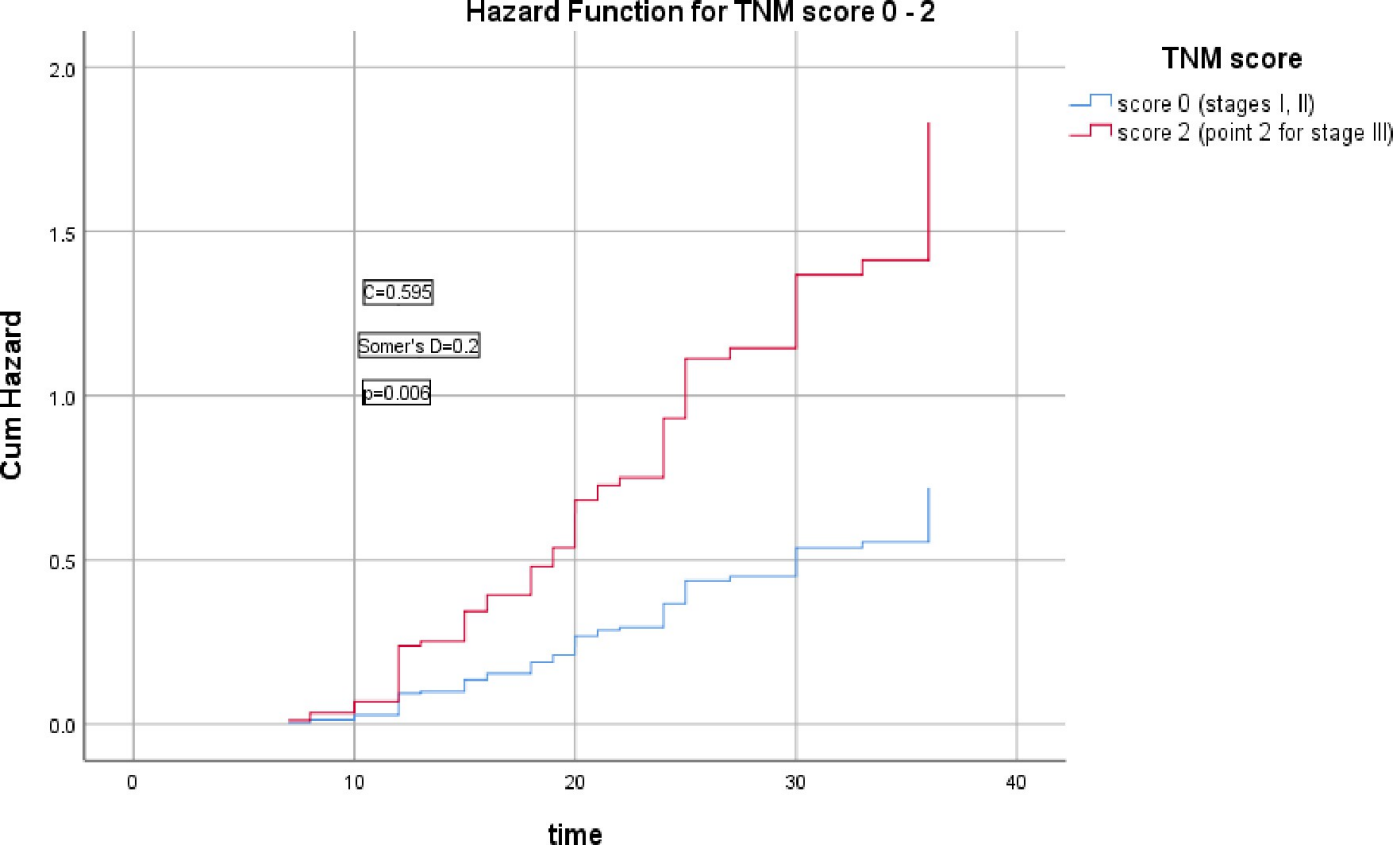
Hazard functions for cTNM model and its scoring showed the highest risk of LRR was for stage III (score 2), each score had only one point but stages I, II did not had significant HR of LRR so pointed as 0, while stage III had a significant HR of LRR and pointed 2.

**Fig 7:**
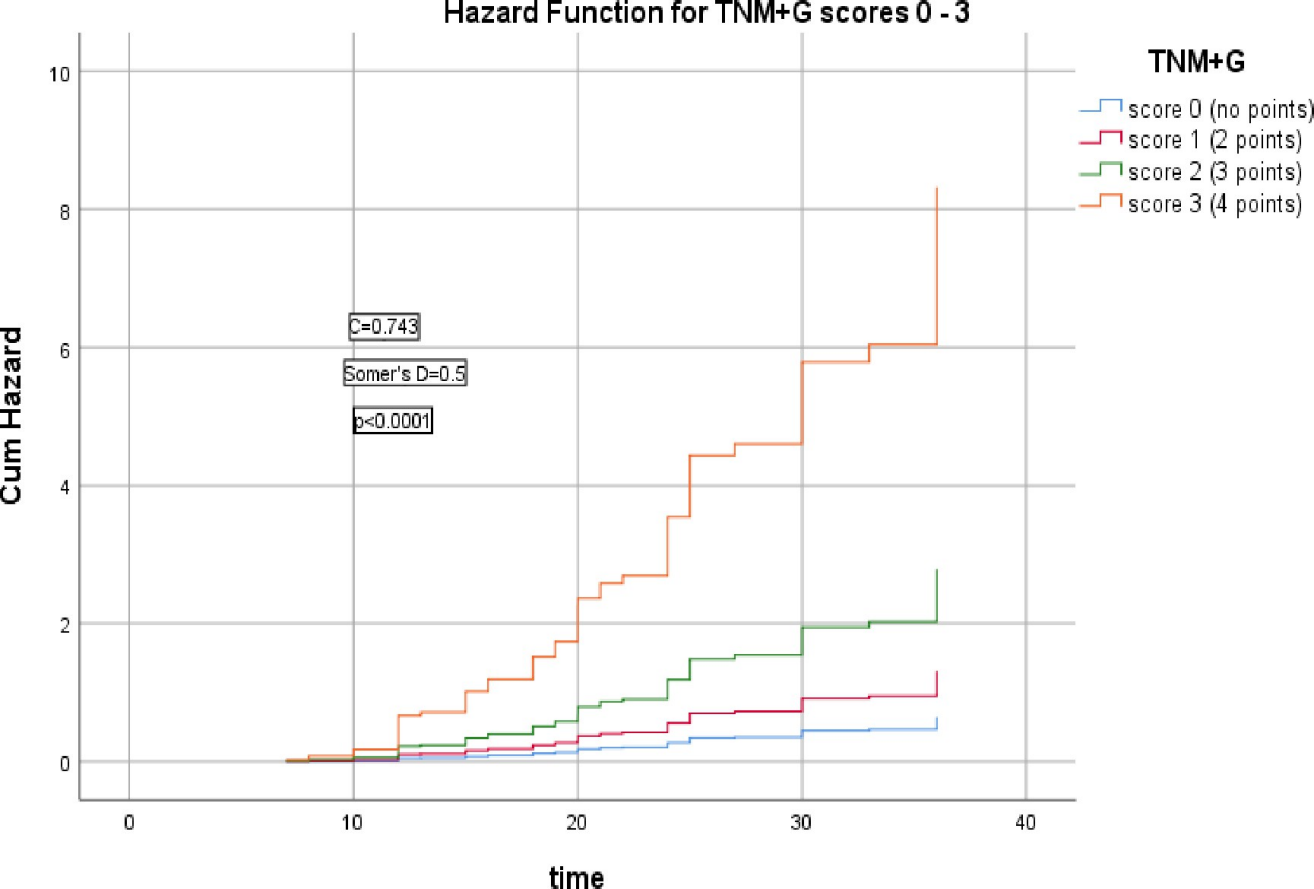
Hazard functions for cTNM + G model and its scoring showed the highest risk of LRR was for score 3.

**Fig 8:**
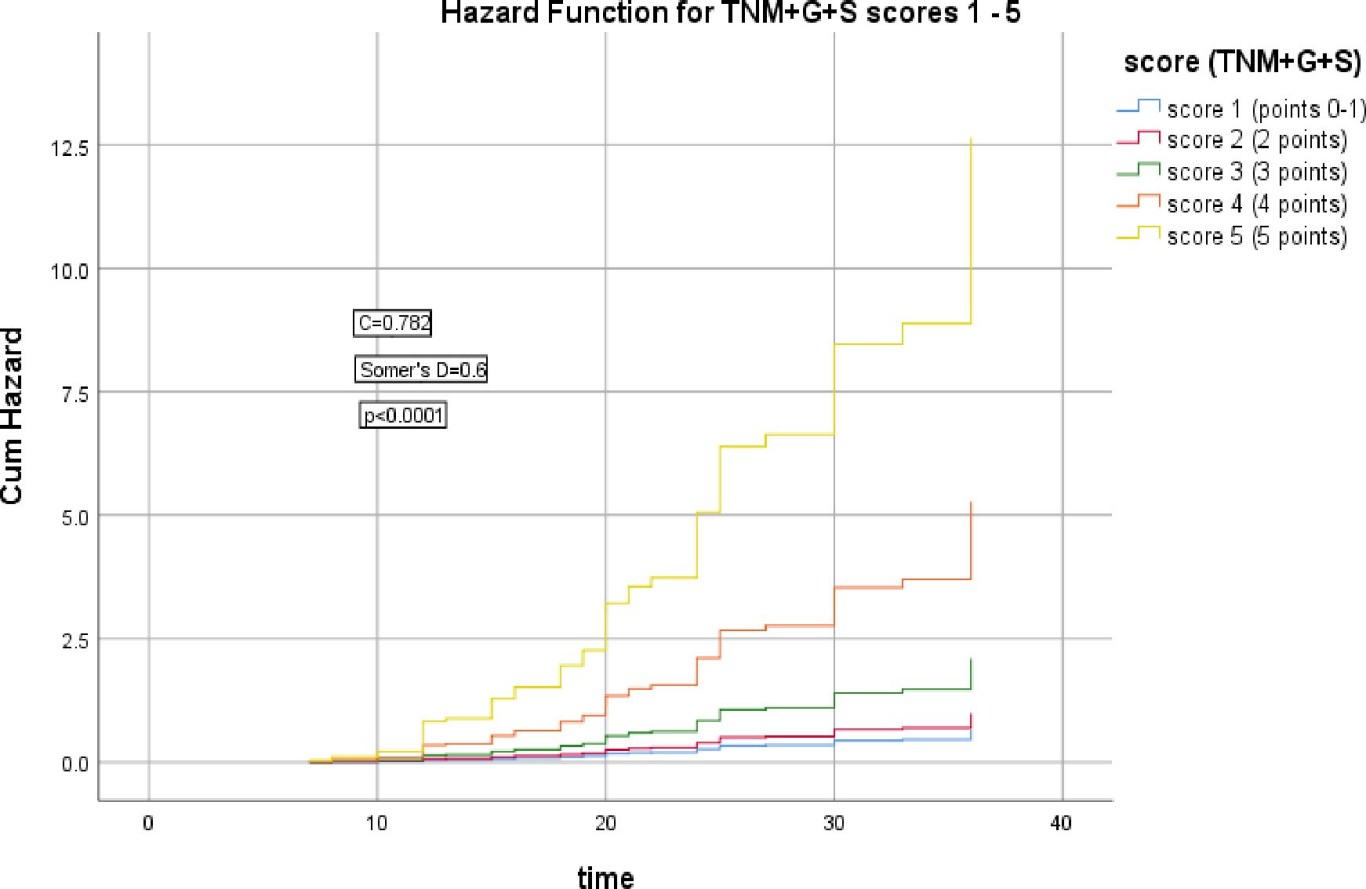
Hazard functions for TNM+G+S model and its scoring showed the highest risk of LRR was for score 5.

**Fig 9:**
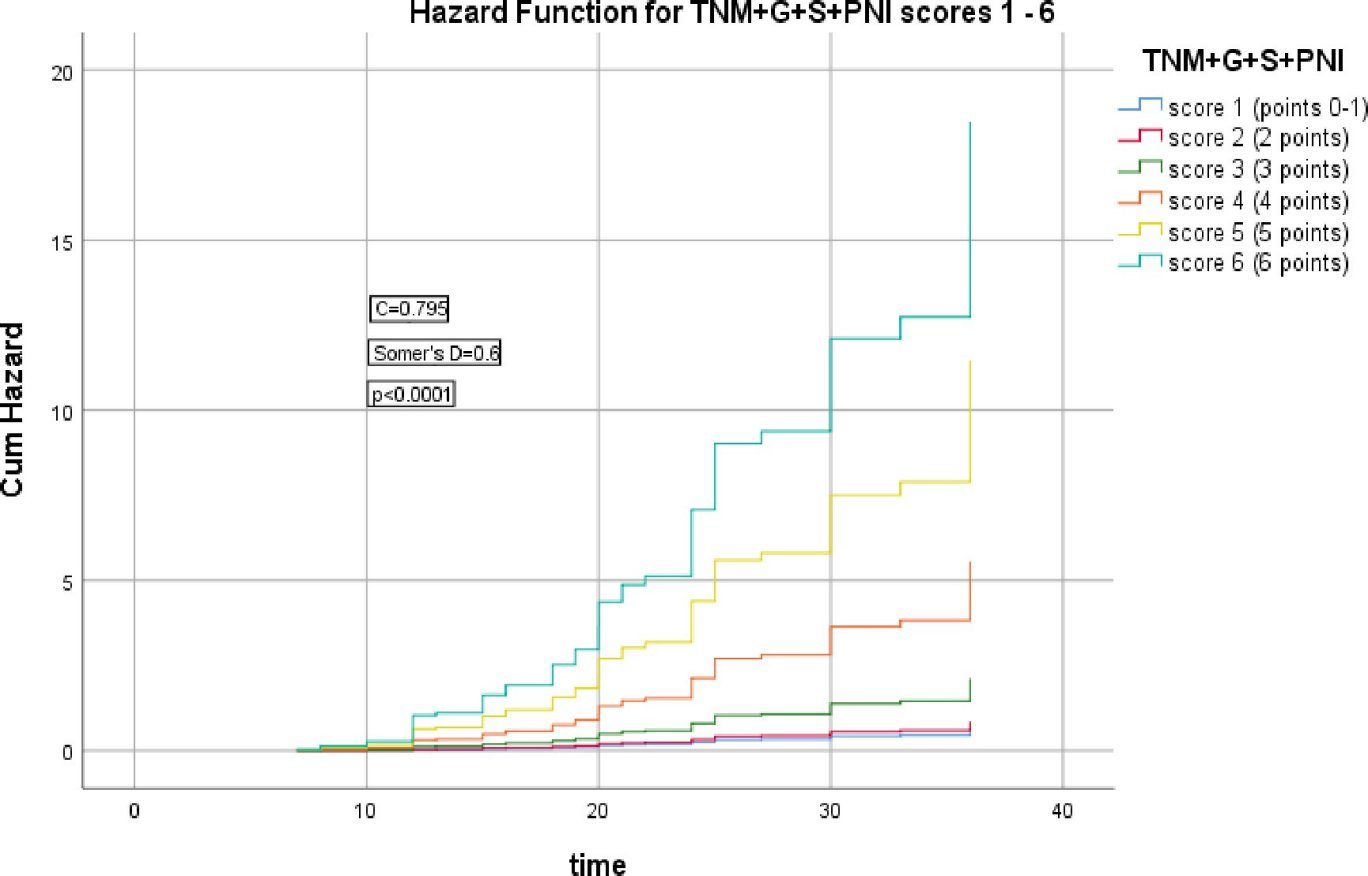
Hazard functions for TNM+G+S model and its scoring showed the highest risk of LRR was for score 6.

**Fig 10:**
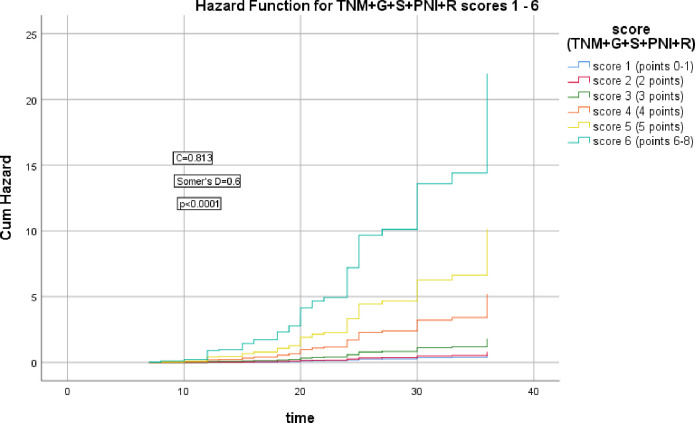
Hazard functions for TNM+G+S+PNI+R model and its scoring showed the highest risk of LRR was for score 6.

The 2^nd^ group of models including cTNM stage, cTNM+G, cTNM+G+S, cTNM+G+S+PNI, cTNM+G+S+PNI+R scores which were significantly correlated with the HR of LRR (Somer’s D = 0.2, 0.5, 0.6, 0.6, & 0.6 respectively), (*p* = 0.006, <0.0001, <0.0001, <0.0001, <0.0001 respectively), the corresponding Harrell’s C indices were 0.595, 0.743, 0.782, 0.795, & 0.813 and AIC were 435.186, 391.683, 371.738, 365.679, 348.375 respectively, the last model had the highest C index and lowest AIC, as shown in Figs 6–10.

The estimated 3-year RFS for cTNM scores ranged from 20.5% to 70.6%, while that of cTNM+G+S+PNI+R ranged from 0%-91.7%. the estimated 3-year RFS for T+N scores ranged from 0%-76.2%, while that of T+N+G+S+PNI+R was 0%-88.2% as reported in Tables 4 and 5.

**Table 4:** TNM stage and its corresponding neoscore and 3-year RFS.

Predictor	% of 3-year RFS	Neoscore	% of 3-year RFS
1-cTNM stage			
I	5/6 (83.3%)	Score 0	12/17 (70.6%)
II	7/11 (63.6%)		
III	17/83 (20.5%)	Score 2	17/83 (20.5%)
Total	29/100 (29%)		
2-Pathologic stage		T+N score	
1	5/6 (83.3%)	Score 1	16/21 (76.2%)
2A	7/10 (70%)	Score 2	11/42 (26.2%)
3A	11/16 (68.75%)	Score 4	0/11 (0%)
3B	5/45 (11.1%)	-----	
3C	1/23 (4.3%)	-----	

Data analyzed using crosstabs and expressed as percentage.

**Table 5:** The estimated 3-year RFS for T+N+G+S+PNI+R and TNM+G+S+PNI+R.

T+N+G+S+PNI+R	3-year RFS	TNM+G+S+PNI+R	3-year RFS
Score 1	15/17 (88.2%)	Score 1	11/12 (91.7%)
Score 2	7/12 (58.3%)	Score 2	13/23 (56.5%)
Score 3	6/21 (28.6%)	Score 3	5/23 (21.7%)
Score 4	1/22 (4.5%)	Score 4	0/12 (0%)
Score 5	0/14 (0%)	Score 5	0/12 (0%)
Score 6	0/10 (0%)	Score 6	0/18 (0%)
Score 7	0/4 (0%)	-----	-----

Data analyzed using crosstabs and expressed as percentage

## Discussion

The most intriguing and object of interest to many researchers and the aim of many ongoing studies is to find strategies to identify prognostic and predictive markers of the outcome for patients with colorectal cancers. The extreme heterogeneity in the survival rates that results from different clinical trials could be attributed wide range of patients’ characteristics and prognostic factors [21].

The 7^th^ TNM classifications of cancer rectum provide a lot of information gathered into different prognostic groups and anatomic stages [22], each combination of T and N in this classification is translated into different impacts on survival, risk of local recurrences and distant metastasis.

However, data regarding grade, site of the tumor in the rectum, pathologic subtype, pretreatment CEA, PNI, LVI and response to neoadjuvant treatment, in addition to RAS-BRAF status, microsatellite instability and EGFR mutations are not part of staging stratifications in spite of being clinically relevant to treatment outcomes, and different treatment guidelines relay dependently on their statuses.

We tried in the current retrospective cohort to illustrate that incorporating the pre-mentioned predictive and prognostic markers were more precise with respect to determining RFS than pathologic stage alone in locally advanced rectal cancer, our results showed that the estimated 3-year RFS for T+N+G+S+PNI+R model ranged from 0–88.2% and Harrell’s C index of 0.843 instead of 0–76.2%, and C index of 0.751 for T+N alone, while cTNM+G+S+PNI+R model was associated with RFS from 0–91.7%, and C index of 0.813 instead of 20.5%-70.6%, and C index of 0.595 for cTNM stage alone indicating that adding other prognostic and predictive markers to pathologic stage or cTNM improved the model goodness and correlated better with 3-year RFS.

It is established that patients with good prognosis are selected for primary surgery with 5-year survival rates approaching 90% [23], including patients with circumferential radial margin (CRM) >1mm, T1-2 lesions, N0-1, tumors >6 cm from anal verge, and no extramural vascular invasion [24], while patients with T3-4, N2 disease, CRM<1mm, tumors <6cm from anal verge, or extramural vascular invasion are associated with significant risk of local recurrences [23, 24], subsequently the role of neoadjuvant treatment.

Current recommendations for rectal cancer consider performing endorectal ultrasonography or MRI for local staging, combined with CT of the chest, abdomen and pelvis for searching of distant metastases [25, 26], to accurately determine patients greatly benefit from neoadjuvant CRT that reduced local recurrences from 25%-40% to less than 10% [27, 28], furthermore, Sauer et al. [29] have shown a reduction in the rate of local recurrence in patients receiving preoperative CRT compared with those who received postoperative CRT (5-year local recurrence of 6% versus 13%, respectively).

Bioscore was adopted in breast cancer incorporating the staging system and several biologic factors done on 3,728 patients treated with surgery between 1997 and 2006; these factors include grade and estrogen receptor status and proved to be more impactful in determining (disease specific survival) DSS [30]. Moreover, neo-Bioscore in breast cancer, which combines the presenting clinical stage, final pathologic stage, grade, ER status and HER2 status to provide more prognostic information for patients receiving neoadjuvant chemotherapy [19].

Data regarding applicability and validity of bioscore in other solid malignancies are lacking, so we thought that this study was the first one to address this issue, although it was a small cohort of patients and we tried to apply a score in a similar way to Bioscore in breast cancer [18], our results found that cTNM stage was associated with 20.5%-83.3% 3-year RFS, which were higher than the corresponding scores, in a similar way pathologic stage was associated with higher 3-year RFS than its corresponding scores which weren’t in alignment with that calculated in Neo-Bioscore of breast cancer.

In addition, patients with cTNM+G+S+PNI+R score >3 did not gain any 3-year RFS benefit compared to those with T+N+G+S+PNI+R score >4 who in turn did not gain any 3-year RFS benefit.

Patients with cTNM+G+S+PNI were associated with 3-year RFS of 0%-80% which further improved by the addition of response to neoadjuvant CRT to 0%-91.7%, however, patients with score >3 did not gain any survival advantage.

This study was an early one to evaluate the applicability of similar scores to bioscore in breast cancer over a small cohort of patients in our department, in spite, if applied on further large cohorts of multiple oncology centers could configure the final prognostic model for the recurrences following CRT.

## Conclusion

We propose that the addition of biologic factors to staging of rectal cancer provide precise stratification and association with local recurrences in patients received preoperative CRT, moreover depending on the anatomic extent of the tumor limits the possibility to fully understand the prognosis and properly make the treatment decisions.
